# Enhancing Mixed Traffic Flow with Platoon Control and Lane Management for Connected and Autonomous Vehicles

**DOI:** 10.3390/s25030644

**Published:** 2025-01-22

**Authors:** Yichuan Peng, Danyang Liu, Shubo Wu, Xiaoxue Yang, Yinsong Wang, Yajie Zou

**Affiliations:** 1 Key Laboratory of Road and Traffic Engineering of Ministry of Education, Tongji University, Shanghai 201804, China; yichuanpeng@tongji.edu.cn (Y.P.); shubowu@tongji.edu.cn (S.W.); yajiezou@hotmail.com (Y.Z.); 2 CAAC Key Laboratory of General Aviation Operation, Department of General Aviation, Civil Aviation Management Institute of China, Beijing 100102, China; xiaoxue_yang@hotmail.com; 3 Nebula Link (Shanghai) Technology Co., Ltd., Shanghai 201804, China; wangyinsong@nebula-link.com

**Keywords:** connected and autonomous vehicles, microscopic simulation, traffic operation, traffic safety

## Abstract

As autonomous driving technology advances, connected and autonomous vehicles (CAVs) will coexist with human-driven vehicles (HDVs) for an extended period. The deployment of CAVs will alter traffic flow characteristics, making it crucial to investigate their impacts on mixed traffic. This study develops a hybrid control framework that integrates a platoon control strategy based on the “catch-up” mechanism with lane management for CAVs. The impacts of the proposed hybrid control framework on mixed traffic flow are evaluated through a series of macroscopic simulations, focusing on fundamental diagrams, traffic oscillations, and safety. The results illustrate a notable increase in road capacity with the rising market penetration rate (MPR) of CAVs, with significant improvements under the hybrid control framework, particularly at high MPRs. Additionally, traffic oscillations are mitigated, reducing shockwave propagation and enhancing efficiency under the hybrid control framework. Four surrogate safety measures, namely time to collision (TTC), criticality index function (CIF), deceleration rate to avoid a crash (DRAC), and total exposure time (TET), are utilized to evaluate traffic safety. The results indicate that collision risk is significantly reduced at high MPRs. The findings of this study provide valuable insights into the deployment of CAVs, using control strategies to improve mixed traffic flow operations.

## 1. Introduction

With the development of autonomous driving technology, it is certain that connected and autonomous vehicles (CAVs) and human-driven vehicles (HDVs) will coexist on the roads within traffic systems for the foreseeable future. On the one hand, CAVs can enhance traffic efficiency and reduce energy consumption and emissions to a certain extent. On the other hand, the principles of traffic operation have changed due to the differences in driving behaviors between HDVs and CAVs, introducing potential traffic risks. Therefore, it is essential to comprehensively assess the impacts of CAVs on mixed traffic operations [1,2,3].

To investigate the impacts of CAVs on the operation of traffic flow, most previous studies primarily utilize two approaches: theoretical analysis and microscopic simulation [4,5,6]. The theoretical analysis always assumes an idealized traffic condition and uses mathematical formulas to deduce the traffic operation state. However, due to the constrained number of parameters, this approach fails to adequately describe real-world complex traffic conditions. The microscopic simulation provides another perspective for analyzing traffic operation characteristics. It can replicate complex traffic phenomena by modeling driving behavior, thereby facilitating operational analysis within mixed traffic flows. Microscopic simulation has been extensively used to analyze the operational characteristics of mixed traffic flow, such as capacity, safety, and stability.

Many previous microscopic simulation studies investigated the impact of CAV integration on road capacity. Generally, the integration of CAVs allows traffic flow to maintain a relatively small time headway, which can enhance road capacity. Shi and Li [7] noted that while short headways can significantly improve traffic capacity, inappropriate headway settings can adversely affect traffic flow stability and reduce traffic capacity. A significant finding is that the traffic capacity significantly increases when the MPR of CAVs reaches moderate or high levels (e.g., above 40%) [8,9]. Shang and Stern [10] investigated the relationship between stability and traffic capacity, revealing that bottleneck capacity is significantly influenced by string stability and time headway. For platoon control strategies, several studies have verified that an appropriate platoon size can enhance both traffic capacity and stability [11,12]. Sala and Soriguera [13] proposed a macroscopic modeling approach to estimate the capacity of freeway lanes under mixed traffic conditions with CAV platoons. Their study demonstrated that optimizing platoon lengths based on traffic demand and CAV penetration rates can significantly improve freeway capacity. Zhou and Zhu [14] revealed that while maximizing platoon size can improve traffic capacity, it is detrimental to the stability of mixed traffic flow. This study determined that an optimal platoon size of four vehicles effectively balances the enhancement of traffic capacity with the maintenance of traffic stability. According to Wang et al. [15], a maximum platoon length of eight provides the optimal balance for achieving the best overall performance in terms of comprehensive effectiveness. Woo and Skabardonis [16] developed a strategy for platoon formation based on traffic conditions, which demonstrated in highway simulation scenarios that this strategy enables CAVs to successfully form longer platoons while ensuring maximal traffic flow. Sakaguchi et al. [17] demonstrated that the real-time optimization of CAV trajectories can significantly improve traffic flow efficiency, fuel economy, and driving safety through the effective coordination of lane changes and merging behaviors. Regarding dedicated lane control, Talebpour et al. [18] evaluated the impact of using dedicated lanes for CAVs on traffic flow and concluded that traffic capacity increased significantly when the MPR exceeded 30%.

Previous studies have demonstrated that minor disturbances in traffic flow can accumulate and propagate upstream, leading to traffic flow oscillations on highways [19,20]. Through microscopic simulation, the traffic oscillation phenomenon can be accurately reproduced, allowing for the derivation of significant conclusions regarding traffic dynamics and stability. Zheng et al. [21] utilized wavelet transform to simulate traffic oscillations, uncovering patterns and periodic fluctuations within traffic flow. From the perspective of shockwave dynamics, Schakel et al. [22] identified a significant correlation between the propagation of dynamic traffic jams and the MPR of CAVs. This study suggested that as the MPR exceeded 50% in mixed traffic, the shockwaves experienced a reduction in both their propagation range and spreading speed, leading to a dampening effect on traffic oscillations. Similarly, Talebpour and Mahmassani [23] investigated the impacts of varying MPRs on traffic stability through simulation. The result demonstrated that increasing the MPR of CAVs significantly enhances traffic stability by preventing the formation and propagation of shockwaves. In addition to MPR, some studies have explored other factors influencing mixed traffic flow stability. For instance, Sun et al. [24] analyzed the interaction between car-following instability and traffic oscillations, emphasizing that the desired time headway is crucial in mitigating traffic shocks. Factors such as speed conditions, the uncertainty of HDV driving behavior, and substantial communication delays in CAVs also significantly impact traffic oscillations [25,26,27,28].

For traffic safety, surrogate safety measures (SSMs), including time to collision (TTC) and post-encroachment time (PET), have been employed to assess the traffic risk [29,30]. An interesting finding is that conflict risk trends among different road segments are inconsistent at MPRs. However, at high MPRs, CAVs significantly reduce traffic risks as measured by TTC and PET [31,32]. Zhu and Tasic [33] proposed a merging conflict model to assess the safety of CAVs in freeway on-ramp scenarios. The findings indicate that CAVs can significantly reduce the severity and frequency of conflict events during merging operations. Time exposed time to collision (TET), modified time integrated time to collision (TIT), distributions of acceleration, and speed difference have also been used as indicators to measure traffic safety [34,35,36]. Note that in TET and TIT, inappropriate parameter threshold settings can increase collision risk and even lead to traffic oscillations [35,36]. These studies suggested that there was a significant improvement in traffic safety when the MPR of CAVs increased.

In heterogeneous traffic flow, the coexistence of HDVs and CAVs leads to dynamic and complex interactions, potentially causing traffic disturbances. To mitigate frequent interactions, separating heterogeneous groups is crucial. Common approaches include the use of dedicated lanes and platoon strategies. The dedicated lane is typically positioned in the innermost lane of a multi-lane road, transitioning the CAVs from a stochastic distribution to the dedicated lane with a regular arrangement [37,38]. However, this strategy can lead to the inefficient use of limited road resources, particularly at low MPRs of CAVs. The platoon strategies allow vehicles to flexibly join or leave a platoon [39,40,41]. More specifically, platooning strategies enable CAVs to maintain small time headways, thereby improving traffic efficiency. Gong and Du [42] proposed a cooperative platoon control strategy to suppress traffic oscillation propagation and improve traffic flow stability. Yang et al. [43] developed a catch-up coordination platoon control strategy to assess the operation characteristics of mixed traffic flow.

In summary, most existing studies have primarily focused on either platoon control strategies or dedicated lane control to improve traffic operations. Few studies have considered the impact of vehicle platoon control strategy and dedicated lane management on mixed traffic flow. To address this limitation, this study proposes a CAV hybrid control framework that combines the platoon coordination strategy with dedicated lane management for multi-lane freeway on-ramps. According to this framework, this study conducted simulation experiments to comprehensively investigate the impacts on mixed traffic flow characteristics, including capacity, traffic oscillation, and traffic safety. The contributions of this study are twofold: (1) a hybrid control framework that combines platoon control and dedicated lane management was proposed to improve traffic capacity, reduce traffic oscillations, and enhance traffic safety; (2) a set of simulation experiments on freeway multi-lane on-ramps was conducted to verify the performance of the proposed hybrid control framework on traffic operations. The evaluation focused on the fundamental diagram, the propagation of shockwaves in the spatiotemporal diagram, and traffic conflicts identified by SSMs.

The remainder of this study is presented as follows. Section 2 introduces the simulation details, including the proposed platoon control strategy and the analysis method in this study. Section 3 provides the impacts of the proposed platoon strategy on the traffic flow from the three aspects of fundamental diagram, traffic oscillation, and traffic safety. Finally, Section 4 summarizes the conclusions and future work.

## 2. Simulation Setup and Methodology

### 2.1. Simulation Settings

This study employs the Simulation of Urban Mobility (SUMO) to conduct simulation experiments under three distinct scenarios: base scenario, platoon control scenario [43], and hybrid control framework scenario. The basic scenario and platoon control scenario are specified as the benchmark to evaluate the performance of the proposed hybrid control framework. The hybrid control framework is developed by integrating the platoon control strategy with dedicated lane management when the MPRs are larger than 40%. Note that the basic simulation setup for these three scenarios is identical, with the primary difference being the control strategies employed.

**Figure 1:**
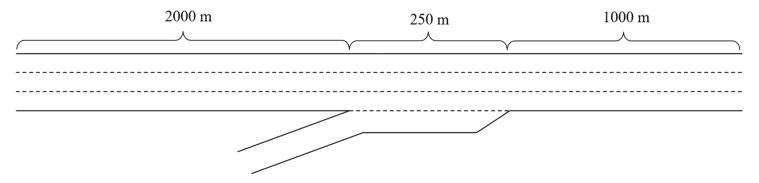
Schematic diagram of the road segment.

The simulation settings are summarized as follows, including road geometry, traffic composition, detector configuration, traffic flow rate, etc. Figure 1 illustrates the experimental roadway segment, which consists of a three-lane freeway and a single-lane on-ramp that frequently acts as a congestion point. This segment spans 3.25 km, with the on-ramp situated 2 km from the starting point and featuring a 250 m long acceleration lane [44,45]. The choice of this 3.25 km freeway segment is driven by the need to stabilize traffic flow over the first 2 km before the on-ramp, and to study the impact of the merging zone on downstream traffic over the following 1 km. The 250 m acceleration lane represents a typical on-ramp length, allowing for realistic vehicle merging dynamics. Traffic metrics such as flow, speed, and density are collected at the on-ramp’s inception every five minutes using two sets of detectors. This study focuses on mixed traffic flow, specifically examining the MPR of CAVs to evaluate the effectiveness of various control strategies. The MPR for CAVs is incrementally varied from 0% to 100%, increasing by 20% at each step. The simulations are conducted over a duration of 3600 s, with each simulation step lasting 0.1 s and incorporating a 300 s initial warm-up period. Data on traffic flow, density, and speed are collected every 5 min from a detector located near the merge area. The settings assume a 1.5 s headway between vehicles, with a baseline demand of 2400 veh/h/lane, increasing by 300 veh/h/lane.

### 2.2. Intelligent Driver Model

The intelligent driver model (IDM) is commonly employed to determine acceleration based on free-flow speed and relative distance, providing a standard approach to describing car-following dynamics. The IDM can be formulated as follows:

(1) $$\hat{a}=a_{m}\left[1-{\left(\frac{v}{v_{d}}\right)}^{4}-{\left(\frac{s*\left(v,∆v\right)}{s}\right)}^{2}\right]$$

where $\hat{a}$ denotes the acceleration of the vehicle, influenced by speed v. $a_{m}$ is maximum desired acceleration, governed by its mechanical capabilities. The term $v_{d}$ denotes the maximum desired speed, which is the highest speed achievable under free-flow traffic conditions. s represents the net inter-vehicle spacing. $s*\left(v,∆v\right)$ indicates the optimal distance between two sequential vehicles, as defined as follows:

(2) $$s*\left(v,\Delta v\right)=s_{0}+\max\left\{0,vT+\frac{v\Delta v}{2\sqrt{a_{m}b_{m}}}\right\}$$

where $s_{0}$ is the minimum safe distance when vehicles are stationary, T denotes the safe time headway, and $b_{m}$ is the comfortable deceleration.

The parameters of the IDM are both realistically interpretable and quantifiable. The IDM not only captures vehicle dynamics in free-flow conditions but also accurately represents car-following behaviors under congested traffic scenarios [46]. Therefore, this study employs the IDM to model the driving behaviors of HDVs. The parameter calibration presented in Table 1 is based on three distinct trajectory datasets using three different deviation measures, as documented by Kesting and Treiber [47].

### 2.3. Cooperative Adaptive Cruise Control Model

The car-following control logic of CAVs used in this study is described using the cooperative adaptive cruise control (CACC) model. Distinct from traditional car-following models, the CACC model can consider a realistic association between vehicle accelerations, relative speeds, and distances. The model parameters are calibrated using empirical trajectory data from field experiments. To address the limitations of the original CACC model, which was effective primarily in high-speed contexts, Xiao et al. [48] refined the model to cover a broader range of speeds by adjusting for varying spacing thresholds.

In the absence of leading vehicles, the control system of CAVs transitions to speed control mode, demonstrated as follows:

(3) $$a_{i,t}=k\left(v_{d}-v_{i,t-1}\right)$$

where $v_{d}$ is the desired speed, $v_{i,t-1}$ denotes the vehicle speed at time t − 1, and k represents the coefficient, which is assigned as 0.4 [48].

The process of controlling the gap is described using a first-order function, where the vehicle speed at time t is computed using the speed at the previous time t − 1, along with the gap deviation $e_{i,t-1}$ and its rate of change ${\dot{e}}_{i,t-1}$:

(4) $$v_{i,t}=v_{i,t-1}+k_{p}e_{i,t-1}+k_{d}{\dot{e}}_{i,t-1}$$

(5) $$e_{i,t}=x_{i-1,t-1}-x_{i,t-1}-d-t_{d}v_{i,t-1}$$

where $k_{p}$ and $k_{d}$ represent the gain coefficients, with values of 0.45 and 0.25, respectively [42]. The term d denotes the vehicle length.

The goal of the gap-closing strategy is to minimize speed differences and reduce the distance between the following vehicles. Therefore, the formula remains consistent with that used in the gap control mode. The sole distinction lies in parameters $k_{p}$ and $k_{d}$, which are set to 0.01 and 1.6.

### 2.4. Hybrid Control Framework

**Figure 2:**
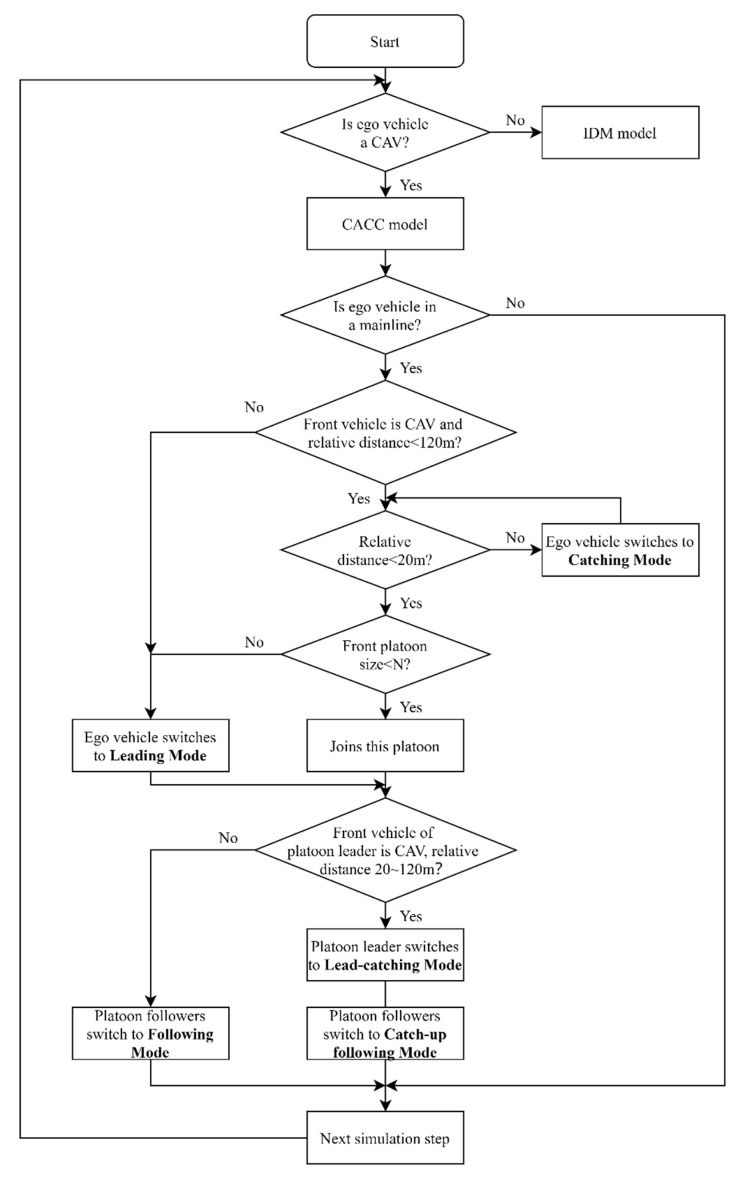
Flowchart for platoon control strategy.

Many studies overlook the complexities involved in the formation process of a platoon, often assuming that vehicles integrate into the platoon immediately without any intermediate stages. This simplification fails to capture the realities of actual driving conditions. To address this issue, this study proposed a hybrid control framework that combines platoon control and dedicated lane management. The dedicated lane for CAVs is deployed in the innermost lane of the simulation road segment when the MPRs exceed 40%. The platoon control strategy is developed based on the “catch-up” mechanism and the consideration of maximum platoon size, as illustrated in Figure 2. Through limited-range vehicle-to-vehicle (V2V) communication, CAVs can switch between two specific driving modes.

Formation of a platoon without rapid approach: If the gap between vehicles is under 20 m, the following CAV merges into the existing platoon ahead, entering following mode. Simultaneously, the vehicle at the front of the platoon adopts leading mode, as depicted in Figure 2.

Platoon mode enabling rapid approach: When the relative distance between a CAV and another vehicle is between 20 m and 120 m and the size of the front platoon is smaller than nine, the CAV enters catch-up mode. In this mode, the CAV accelerates to a higher desired speed to close the gap with the nearest vehicle in the same lane, aiming to merge into the platoon ahead. If the CAV was leading a platoon prior to the switch, the following vehicle in that platoon also transitions to catch-up following mode.

To maintain platoon integrity and stability, vehicles are prohibited from merging into the middle of the platoon through cooperative lane-changing maneuvers. If an HDV forcefully merges, the platoon splits into two smaller platoons to accommodate the gap. Similarly, when a CAV merges in the middle, the platoon temporarily splits, and the merging CAV may rejoin the leading platoon if conditions permit. For lane changes at either end of the platoon, HDVs do not affect platoon integrity, while CAVs either form a new platoon or join the target platoon based on its size. If the platoon size exceeds the predefined threshold, the merging CAV forms an independent platoon. Otherwise, it transitions to the leading or following mode depending on whether it merges at the front or rear, maintaining a suitable distance.

When no preceding vehicle is detected within range, CAVs retain their default single-vehicle mode. To differentiate between various driving modes in this study, desired speeds are adjusted based on a specific speed factor. Field experiments by Shladover, Su, and Lu [8] have defined several optimal time gaps for vehicles equipped with CACC. The parameters for each mode are detailed in Table 2. The proposed “catch-up” mechanism optimizes platoon stability and safety by adjusting two key parameters: headway time and speed factor. In leading mode, a larger headway is used to maintain spatial separation between platoons, with a default speed factor of 1.0. Following mode employs smaller headways and a slightly increased speed factor for efficient velocity adjustments. Lead-catching and catching mode use larger headways and higher speed factors to ensure safety during gap closure. Catch-up following mode further refines speed and headway to stabilize platoons, balancing safety and efficiency with a safe time to collision (TTC) threshold of 3 s.

### 2.5. Surrogate Safety Measures

To comprehensively assess the conflict risk, four surrogate safety measures are selected, including time to collision (TTC), criticality index function (CIF), deceleration rate to avoid a crash (DRAC), and total exposure time (TET) [49]. The TTC measures the time remaining before a collision occurs if two vehicles continue at their current speeds and trajectories, serving as a temporal-based safety indicator. The CIF evaluates the severity of potential conflicts by considering both the likelihood and the consequence of a collision, integrating temporal and spatial elements. The DRAC quantifies the required deceleration for a vehicle to avoid a collision, offering a deceleration-based perspective on traffic safety. The TET quantifies the duration of exposure to risk conditions that could lead to traffic accidents or incidents. The formulas of the four indicators are presented as follows:

(6) $$TTC=\frac{x_{L}-x_{F}-d_{L}}{v_{F}-v_{L}}$$

(7) $$CIF=\frac{v_{F}^{2}}{TTC}$$

(8) $$DRAC=\frac{v_{L}^{2}-v_{F}^{2}}{x_{L}-x_{F}-d_{L}}$$

(9) $$TET=\sum_{i=1}^{N}t_{i}$$

where $x_{L}$ and $v_{F}$ are the position and speed of the leading vehicle, respectively, while $x_{F}$ and $v_{F}$ denote the position and speed of the following vehicle. $d_{L}$ represents the length of the leading vehicle. $t_{i}$ is the exposure time for the *i*-th vehicle and *N* is the total number of vehicles or road users considered in the analysis.

## 3. Simulation Results and Analysis

### 3.1. Fundamental Diagram

**Figure 3:**
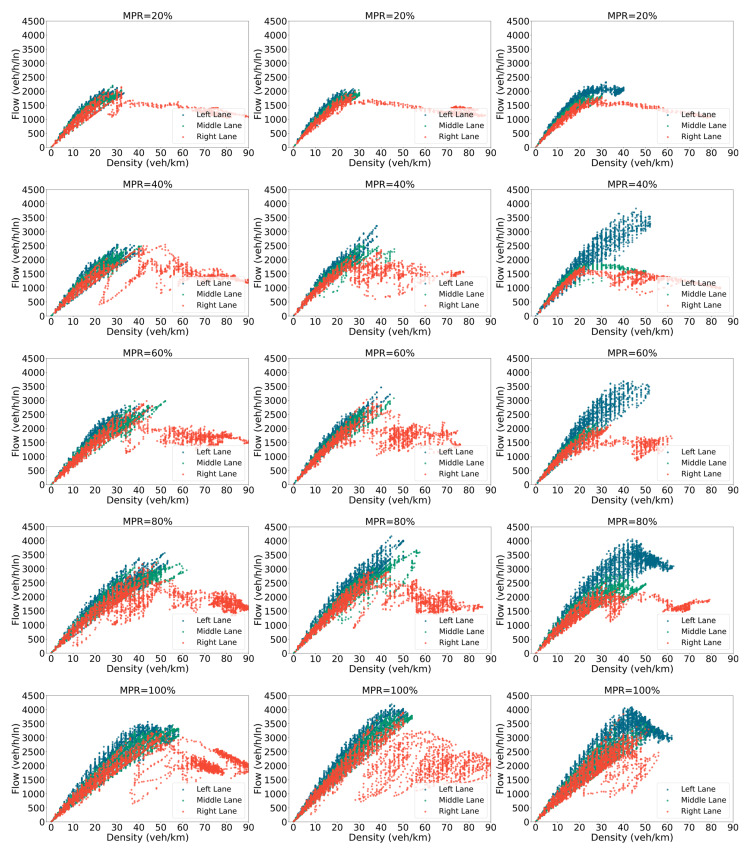
The flow–density diagram.

Figure 3 illustrates the flow–density relationship across various MPRs of CAVs. The triangular shape between flow and density observed in these diagrams for mixed traffic flow is consistent with the findings from previous studies [50,51]. From Figure 3, some interesting findings can be observed. First, as the MPRs of CAVs increase, the flow rate of all three lanes increases. This phenomenon is particularly evident in the dedicated CAV lane (the left lane in the mixed control model). The trends shown in a series of fundamental diagrams indicate that the introduction of CAVs can significantly enhance traffic efficiency. At an MPR of 40%, the traffic flow of the dedicated lane reaches its peak, whereas there is no significant increase in the capacity of the left lane for the other two models. Although more CAVs are allocated to the dedicated lane, resulting in a slightly lower maximum flow in the middle and right lanes under the mixed control model compared to the other two models, the improvements brought to overall traffic efficiency by the dedicated lane in a low to medium MPR environment are still substantial. Secondly, compared to the base scenario and the platoon control scenario, the right lane experiences less congestion at high flow rates in the scenario under the hybrid control framework. This is due to the unlimited platoon size in the platoon control scenario, making it difficult for on-ramp vehicles to merge smoothly. As noted by Hasanzade Zonuzy [52], ensuring appropriate inter-vehicular time gaps and velocities is essential for smooth merging operations in such scenarios. In addition, from the dispersion of data points, it is evident that frequent transitions among various control strategies, such as speed control, gap-closing control, and gap control, contribute to increased heterogeneity in traffic flow. This effect is particularly pronounced as the MPR increases.

**Figure 4:**
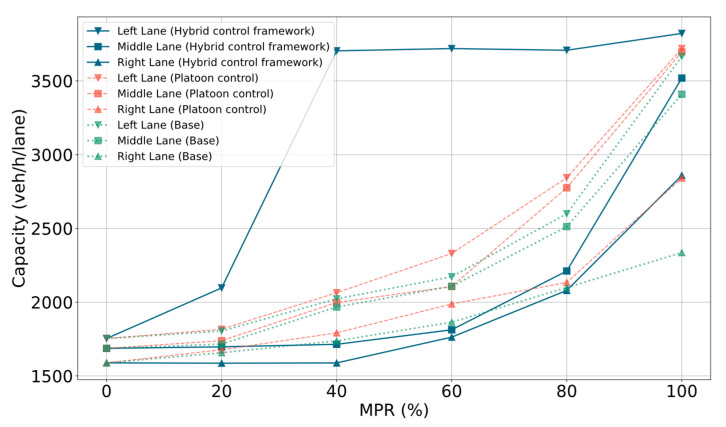
Comparison of road capacity.

This study also examines the impact of various MPRs on freeway capacity across three scenarios. Figure 4 illustrates that road capacity is highly sensitive to changes in penetration rates. Generally, for any control model, the capacity of the three lanes is ranked from highest to lowest as follows: left lane, middle lane, right lane. This is because the right lane is affected by merging behavior from ramp entry lanes, and vehicles in the middle lane nearby are consequently impacted. It can be seen that after implementing a dedicated lane strategy, there is a significant increase in the capacity of the left lane under the hybrid control scenario, reaching its peak at an MPR of 40%. Correspondingly, since the MPR of CAVs in the other two lanes is reduced in this scenario, the capacity of the middle and right lanes is slightly lower than the other two models. However, in terms of overall efficiency, the hybrid control model still shows a notable improvement. Overall, under the platoon control scenario, when the penetration rate reaches 80%, the capacity can increase to 2500 veh/h/lane, a 50% increase compared to a 20% penetration rate. Moreover, in the hybrid control scenario, when the penetration rate reaches 100%, the capacity increases from 3000 veh/h/lane in the baseline scenario to 3300 veh/h/lane. Conversely, in environments with lower penetration rates, where dedicated lane control is not fully implemented, the increase in capacity is less significant.

### 3.2. Traffic Oscillation

**Figure 5:**
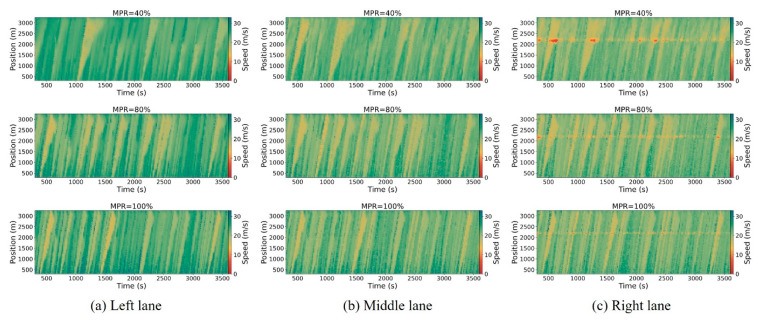
Spatiotemporal trajectory diagrams for the basic scenario.

**Figure 6:**
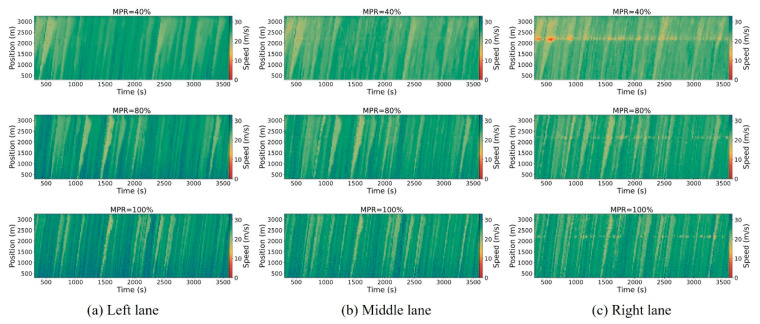
Spatiotemporal trajectory diagrams for the platoon control scenario.

**Figure 7:**
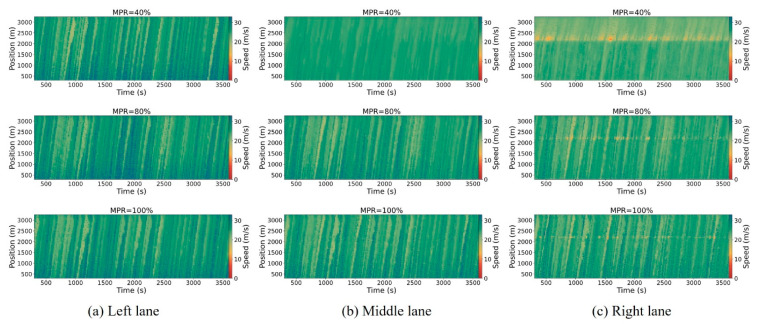
Spatiotemporal trajectory diagrams for the hybrid control framework scenario.

Figure 5, Figure 6 and Figure 7 illustrate the spatiotemporal trajectory diagrams for the three scenarios, offering a clear representation of overall traffic dynamics on the road. In these diagrams, the horizontal axis represents time, the vertical axis represents position along the road, and the color gradient indicates vehicle speed, providing an intuitive understanding of traffic flow and speed distribution over time and space. This study employs spatiotemporal trajectory diagrams to investigate shockwaves caused by the stop-and-go phenomenon in mixed traffic conditions. These diagrams utilize color coding to represent vehicle speeds, providing a clear visualization of traffic dynamics. To ensure clarity in the analysis, the initial warm-up period from 0 to 300 s is excluded.

From Figure 5, Figure 6 and Figure 7, it can be observed that as the MPR of CAVs approaches 40%, there is no significant improvement in mitigating shockwave propagation within the hybrid control framework scenario compared to the base scenario. This is because the traffic flow is predominantly composed of HDVs. However, beyond the 80% MPR threshold, there is a marked reduction in shockwave intensity under the hybrid control framework scenario. Furthermore, vehicle speeds significantly increase as the MPR rises from 80% to 100% in the right lane. In summary, the hybrid control framework that combines platoon control and dedicated lane management in this study effectively reduces traffic oscillations.

The temporal–spatial diagrams of the right lane illustrate the directional patterns and extent of shockwave propagation triggered by stop-and-go behaviors. It can be observed that there is a distinct reduction in the extent of upstream shockwaves, while the extent of downstream shockwaves remains unchanged, fluctuating around 2200 m near the freeway merging area. The upstream boundary of shockwave propagation exhibits a consistent pattern across the three scenarios. At lower MPRs of CAVs less than 20%, there is a modest improvement in the spread of upstream waves. As the MPR increases to 80%, there is a significant enhancement in upstream spread.

### 3.3. Traffic Safety Evaluation

**Figure 8:**
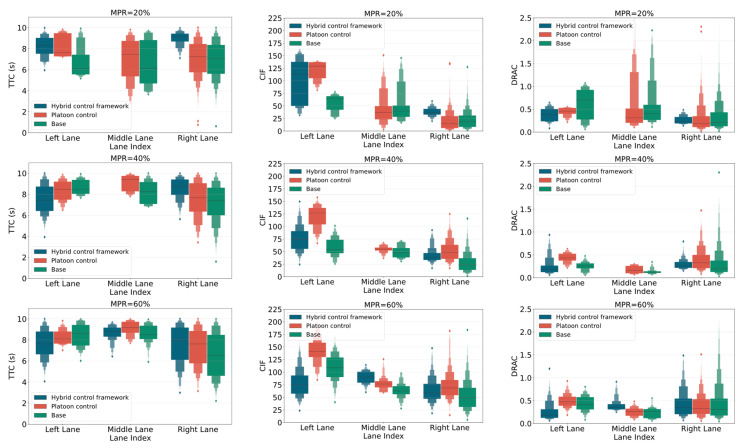

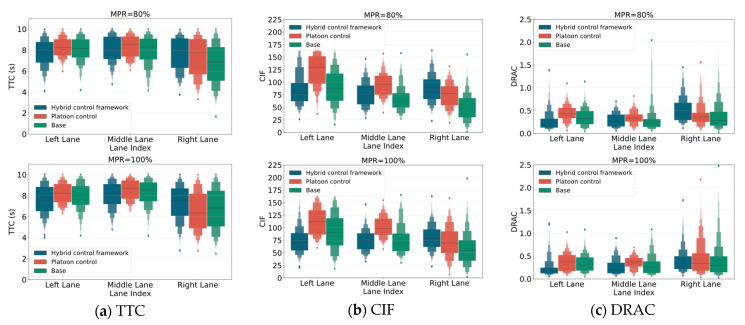
Distributions of TTC, CIF, and DRAC under various MPRs of CAVs.

For traffic safety assessment, four SSMs, namely TTC, CIF, DRAC, and TET, were selected to evaluate traffic safety levels. Figure 8 illustrates the distributions of three metrics across various MPRs of CAVs in three simulation scenarios. Note that the range of TTC is set between 0.5 and 10 s [53], as values below 0.5 s indicate an imminent collision risk, while those above 10 s are considered safe and less critical for analysis. This suggests that there are no observable distributions of TTC, CIF, and DRAC in the middle lane at 20% and 40% MPRs when using the hybrid control framework. This also demonstrates higher safety in the middle lane under these traffic conditions. Figure 8 uses enhanced boxplots, similar to violin plots, to depict both the distribution and density of safety indicators, providing a comprehensive view of their variability and concentration. According to Figure 8, in both the hybrid control framework and platoon control scenarios, the incidence of CIF conflicts rises with an increasing MPR of CAVs, particularly in the middle and right lanes. Similarly, the DRAC measure shows a consistent upward trend as the MPR of CAVs increases.

According to the distribution trends for both the TTC and DRAC in the right lane across three scenarios, it is observed that the base scenario exhibits a higher incidence of conflicts compared to platoon control and hybrid control scenarios when the MPR of CAVs exceeds 40%. Conversely, the CIF distribution shows that the hybrid control framework has lower conflict values than the platoon control in the left lane at various MPRs. This suggests that the hybrid control framework, which combines dedicated lane management, can effectively mitigate conflicts in this lane compared to the platoon control. In summary, for the left and middle lanes, the occurrence of more severe conflicts is more frequent under the platoon control and hybrid control framework. This reflects a global trend where platoon control strategies, despite their benefits in certain conditions, may lead to increased conflict frequencies in these lanes, highlighting the need for optimized control measures to ensure safety across all traffic lanes.

**Figure 9:**
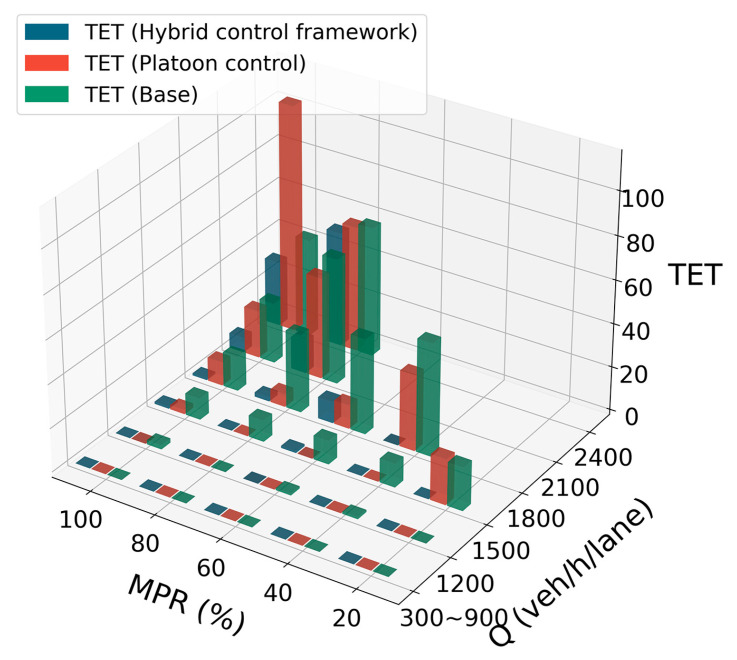
Distributions of TET under various MPRs and traffic flow levels.

Some interesting phenomena are observed in Figure 9. In scenarios with lower traffic flow, the TET values for all three control models are zero. However, for moderate traffic flow, both the mixed control and baseline scenarios show a decreasing trend in safety as the traffic flow increases for the same MPR level. Additionally, for the same traffic flow level, increasing the MPR results in improved safety for both scenarios. For the platoon control scenario, when the MPR remains unchanged and the traffic flow increases, the TET values similarly show a decline. Notably, in higher traffic flow conditions, if the MPR increases, a pattern of initially increasing and then decreasing TET is observed. As shown in Figure 9, this characteristic is evident when the single-lane flow reaches 1800 veh/h/lane. Furthermore, at 2400 veh/h/lane, the high TET values in the platoon control scenario become more pronounced at higher MPRs. Overall, in most scenarios, the TET values for the hybrid control model framework are lower than the other two models, demonstrating an enhancement in terms of safety through the hybrid control framework.

## 4. Conclusions

To comprehensively examine the impacts of CAVs on mixed traffic flow, a hybrid control framework integrating platoon control and dedicated lane management has been developed to leverage the strengths of CAVs. A set of microscopic simulations was conducted at a multi-lane freeway bottleneck with an on-ramp to evaluate the impacts on traffic operation from aspects of the fundamental diagram, traffic oscillation, and traffic safety. According to the simulation results, the main conclusions are provided as follows:

(1) There are significant differences in flow–density plots under varying MPRs across the three scenarios. In more detail, road capacity increases with higher MPRs. Moreover, there is a notable improvement in road capacity under the hybrid control framework compared to the other two scenarios, especially for the dedicated lane.
(2) The proposed hybrid control framework significantly improves traffic efficiency, particularly at higher MPRs. The deployment of dedicated lane and platoon control can mitigate shockwave propagation, reduce traffic oscillations, and improve traffic speed.
(3) In terms of traffic safety, an increase in MPRs of CAVs is associated with a higher collision risk, as identified by the TTC, CIF, DRAC, and TET measures. In addition, compared to the base and platoon control scenarios, the collision risk is lower under the hybrid control framework. This suggests that the deployment of the hybrid control framework can effectively improve traffic safety under high MPRs of CAVs [54,55,56].

While this study highlights the benefits of the hybrid control framework, certain limitations remain. This analysis does not account for scenarios with multiple CAV-dedicated lanes, which could offer further insights into the framework’s impact. Additionally, the effect of varying platoon sizes and intensities has not been considered, potentially influencing the results. Lastly, this study focuses solely on car-type vehicles, excluding trucks, which limits the applicability of the findings to mixed traffic conditions. Future research will address these limitations to enhance the robustness and generalizability of the conclusions. The findings of this study provide valuable insights into the deployment of CAVs, using control strategies to improve mixed traffic flow operations.

## Figures and Tables

**Table 1:** The parameters of IDM.

Parameter	Values	Units
Maximum desired speed $v_{d}$	110	km/h
Safe time headway T	1.5	s
Minimum safe distance $s_{0}$	2.0	m
Comfortable deceleration $b_{m}$	2.0	$m/s^{2}$
Maximum desired acceleration $a_{m}$	1.4	$m/s^{2}$

**Table 2:** Parameters for different modes.

Control Mode	Desired Time Headway (s)	Speed Factor
Leading mode	1.1	1.0
Following mode	0.6	1.1
Lead-catching mode	0.7	1.2
Catch-up following mode	0.6	1.3
Catching mode	0.7	1.2

## Data Availability

The data presented in this study are available on request from the corresponding author. The data are not publicly available due to privacy.
